# Getting real *clean*: a virtual reality training pilot study for cleaning and low-level disinfection of portable medical equipment

**DOI:** 10.1017/ice.2025.89

**Published:** 2025-08

**Authors:** Esteban A. Barreto, Michelle S. Jerry, Vianelly García, Chloe V. Green, Andrea S. Greenfield, Eileen F. Searle, Erica S. Shenoy

**Affiliations:** 1 Division of Infectious Diseases, Massachusetts General Hospital, Boston, MA, USA; 2 Harvard Medical School, Boston, MA, USA; 3 Center for Disaster Medicine, Massachusetts General Hospital, Boston, MA, USA; 4 Department of Medicine, Massachusetts General Hospital, Boston, MA, USA; 5 Infection Control, Massachusetts General Hospital, Boston, MA, USA; 6 Infection Control, Mass General Brigham, Somerville, MA, USA

## Abstract

Portable medical equipment (PME) is inconsistently cleaned and disinfected, resulting in contamination that increases the risk of healthcare-associated infections. A virtual reality PME cleaning and disinfection training module was designed and tested at multiple healthcare facilities. Barriers identified during an initial phase led to improvements in the second phase.

## Background

Maintaining a clean healthcare environment is critical to ensuring patient and healthcare personnel (HCP) safety and reducing risk of healthcare-associated infections (HAI). This includes cleaning and low-level disinfection (LLD) of non-critical portable medical equipment (PME). PME is ubiquitous in healthcare but is inconsistently cleaned, with contamination rates between 25% and 100%; contributing factors include HCP’s limited understanding of its role in HAI transmission and lack of targeted education.^1^ As an emerging training modality for infection prevention and control (IPC), virtual reality (VR) has been implemented to train on performing hand hygiene,^2^ sharps injury prevention among medical and nursing trainees,^3^ and correct use of PPE,^4^ demonstrating improved performance, self-efficacy, and engagement in learners.

Systematic reviews of VR training and education report that VR is more effective or at least on par with traditional pedagogical methods;^5,6^ however, challenges remain, including barriers to implementation and defining objective improvement in HCP competency and performance.^7^ Involving end users in the development and refinement of VR content is critical, as users can inform improvements while highlighting technical barriers and negative physical sensations such as motion sickness and dizziness that may detract from learning objectives.^8^ In this study, a VR IPC module to improve HCP knowledge and practice in PME cleaning and LLD was designed and implemented as part of a quality improvement (QI) project, with participant experience, including the barriers and facilitators to VR uptake, incorporated into module refinement.

## Methods

A multi-disciplinary team of subject matter experts developed a VR training module to improve HCP knowledge and practice regarding cleaning and LLD of PME. The module was developed using the EducationXR application (Heizenrader LLC, Salt Lake City, UT) and used with head mounted displays (HMD, Meta Quest 2 and 3, Meta, Menlo Park, CA). The VR IPC module guides users through a simulated inpatient healthcare environment where they learn and apply cleaning and disinfection concepts to two types of PME: a vital sign machine or point of care ultrasound (POCUS).

Using a plan-do-study-act (PDSA) approach, the research team piloted the VR IPC module to assess feasibility and usability at seven healthcare facilities. At each facility, designated site managers received VR training, either in person or by videoconference, conducted by the research team. Site managers recruited participants and conducted 30-minute VR training sessions at their facilities using portable HMDs. In Phase 1 (P1), participants provided feedback on their experience through semi-structured interviews. Participants were queried about their experience with the HMD and navigating the module. Interviews were conducted in English and Spanish via videoconference, lasted approximately 30 minutes, and were recorded and transcribed. Participants received a $50 gift card upon completing an interview.

A senior data analyst (EAB) oversaw the qualitative data analysis; three independent coders (MSJ, CVG, VG) conducted double coding of verbatim transcripts, which consisted of generating phrases to describe relevant quotes.^9^ Inter-rater reliability was manually calculated. Coding discrepancies were documented, discussed, and resolved collaboratively by the coding team, with EAB making final decisions when needed, ensuring a rigorous coding process. A consensus on emerging codes and overarching themes was reached encompassing both technological aspects and module-specific feedback. Interview results from P1 informed revisions to the VR IPC training module and VR implementation resources before beginning Phase 2 (P2).

During P2, unique participants piloted the updated module. Participants in P2 completed a semi-structured interview, as described in P1, or an online survey, receiving a $25 gift card for completion. Surveys were available in English only and took approximately 15 minutes.

The study received Non-Human Subjects Research (NHSR) determination by the MGB Institutional Review Board (2023, ID 863).

## Results

Between January and March 2024 (P1), participants (n = 31) from three healthcare facilities completed the first version of the VR IPC module and semi-structured interviews. Based on interview findings, revisions were made to the module and the implementation resources between April and June 2024. Between July and November 2024 (P2), participants (n = 44) from four additional healthcare facilities completed the revised module and either a semi-structured interview (n = 24) or a survey (n = 20). Participants’ ages ranged from 22 to 66 years, and the majority worked in nursing (43/75; 57.3%) and reported minimal to no prior experience with using VR (65/75; 86.7%) (Supplementary Table 1).

**Figure 1.**
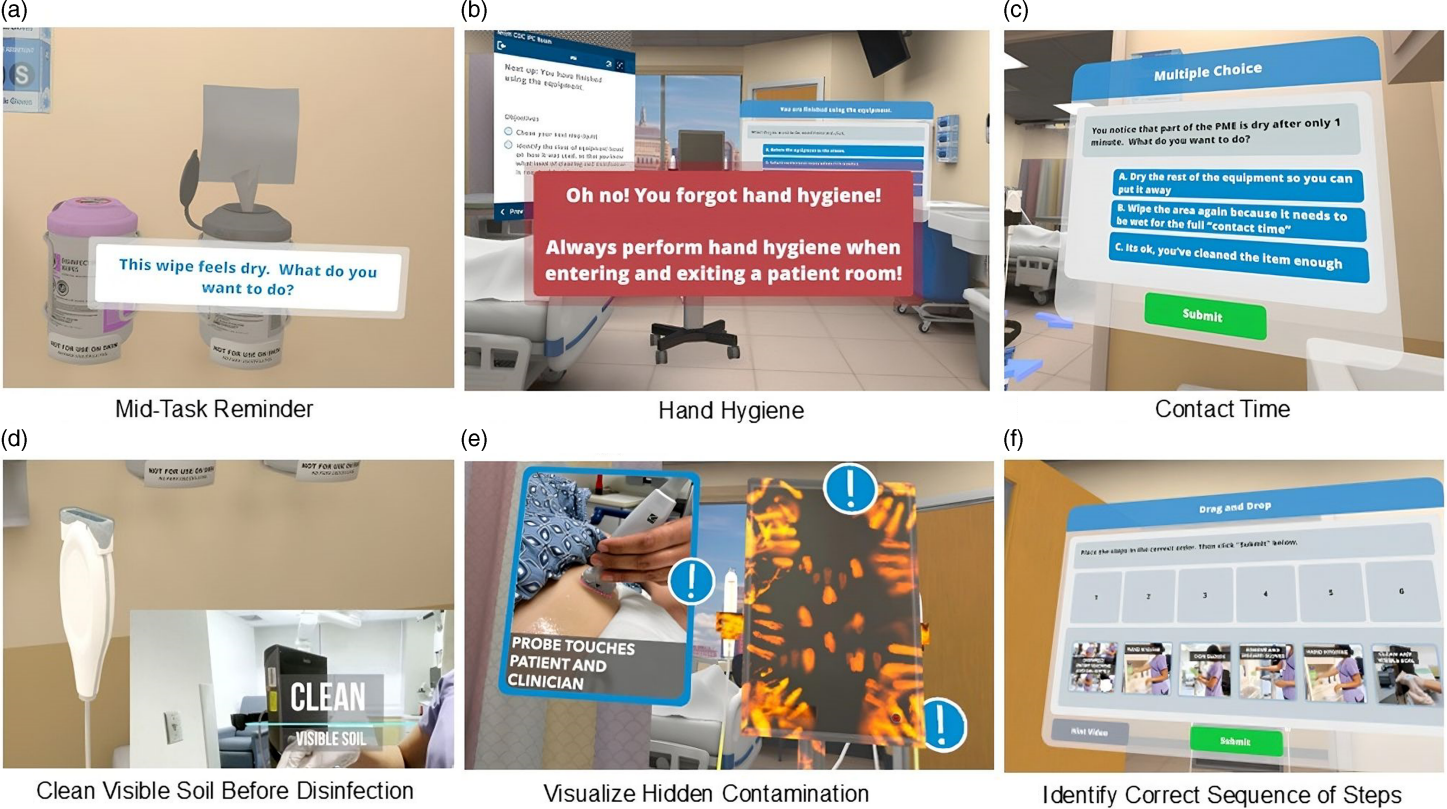
VR scenarios and teaching points. In frame A, user draws a disinfectant wipe from the canister and is prompted that the wipe is dry and asked what they want to do. User must discard the wipe and grab a fresh one to advance. In frame B, if user tries to enter or exit the patient care area without performing hand hygiene (either soap and water wash or alcohol-based hand rub), they will be alerted of the missed step. In frame C, user is alerted that the equipment did not remain wet for full wet time/contact time and is prompted to take action. In frame D, user must wipe visible soil on the transducer, the transducer holder, and the gel bottle. This drives home thoroughness, as well as the importance of cleaning before disinfection. In frame E, user is shown the invisible contamination and can learn about the different ways the equipment can be contaminated. In frame F, user is asked to order the steps correctly to clean and disinfect the machine, including glove donning and doffing and hand hygiene. A video highlighting features of the module is provided (Supplement).

Coding and thematic analysis revealed that most participants in P1 reported an overall positive experience (23/31; 74.2%). Participants praised the high-fidelity environment and the ability to interact with objects in the space. Participants described specific teaching points from the module that were new to them, or valuable review (Figure 1). Negative physical sensations (headaches, eye strain, dizziness, nausea) were reported by 9/31 (29%) and challenges with blurry vision by 17/31 (54.8%). Some participants reported frustration (7/31; 22.6%) and/or fear related to the inability to see their surroundings (12/31; 38.7%). Participants expressed difficulty with the handheld controllers (12/31; 38.7%), transporting the virtual PME (9/31, 29%), and donning and doffing their virtual gloves (8/31; 25.8%). Half of participants (16/31; 51.6%) found the module instructions unclear.

Based on the results from P1, the VR IPC module was revised (Table 1). Technical revisions included: re-programming to permit different controller commands to achieve a similar goal, allowing participants to push multiple buttons and still complete the needed actions; shortening the distance users need to travel with the PME; adding visual indicators for glove interactions; and simplifying instructions. Additional implementation resources were created to address barriers in HMD and training set-up.

**Table 1.** Participant reported barriers and revisions made to address them

Barriers	Phase 1 N = 31 (%)	Phase 2 N = 44 (%)	Selected revisions made between phases
Negative physical sensations	9 (29.0)	20 (45.5)	Minimize text, and supplement with audio. Limited locomotion via joystick.
Blurry vision	17 (54.8)	11 (25.0)	New resources to assist users with glasses in VR.
Difficulty navigating controllers	12 (38.7)	17 (38.6)	Adjusted quiz interactions to allow for both point/click and grab- users could use either.
Bugs	4 (12.9)	5 (11.4)	Debugged hand hygiene door trigger.
Transporting portable medical equipment	9 (29.0)	2 (4.6)	Added instructions on top of the machine on how to transport. Simplified the mechanism of transporting the PME
Difficult glove interactions	8 (25.8)	5 (11.4)	Modified glove interactions for more realism/accuracy.
Unclear or confusing instructions	16 (51.6)	9 (20.5)	Shortened and revised text instructions; added floating help-text in eye-line.

VR, Virtual Reality; PME, Portable Medical Equipment.

Most P2 participants reported an overall positive experience (39/44; 88.6%). Half reported negative physical sensations (20/44; 45.5%), but fewer participants reported module challenges across most domains, such as fewer challenges with transporting PME, glove interactions, and understanding instructions (Table 1).

## Discussion

In this QI project, a VR IPC module was designed and implemented across multiple healthcare facilities. After revisions were made to the module and implementation resources, there was an increase in overall positive experience and a decrease in a few of the reported barriers including less blurry vision, decreased difficulty using the controllers, including when moving the PME and donning and doffing gloves. Across both phases, users reported positive experiences and engagement while exploring a novel mode of learning.

Several limitations are noted. This pilot project included a small sample size with voluntary pilot site and end user recruitment. Participants were mostly in nursing roles, and our sites were mostly technologically resourced facilities in urban settings, so the findings may not be transferable to other healthcare roles, or other settings. Participants were unique to both phases; direct comparisons between the groups related to changes in the VR module are not possible. Most notably, while out of scope for this pilot project, we did not assess the impact of the module on HCP knowledge and competency or compare VR training with traditional IPC education. This project highlights the importance of engaging in pilots and PDSA cycles to refine materials and implementation strategies.

While VR training is increasingly used in medical education, efforts to assess its implementation for training in IPC are few.^8^ This project identified the perceived value of VR IPC training while highlighting technical and implementation concerns, many of which were addressed through modifications based on participant feedback. Future studies will evaluate user knowledge, skills, and competency after the implementation of modifications to mitigate negative physical sensations.

## Supporting information

Barreto et al. supplementary material 1 Barreto et al. supplementary material

Barreto et al. supplementary material 2 Barreto et al. supplementary material

## Supplementary material

To view supplementary material for this article, please visit https://doi.org/10.1017/ice.2025.89
