# Supplementary material for: Getting real clean: a virtual reality training pilot study for cleaning and low-level disinfection of portable medical equipment
Source: Infect Control Hosp Epidemiol. 2025 Jun 11;46(8):852–4. doi: 10.1017/ice.2025.89 (PMC12483617; doi:10.1017/ice.2025.89)
Supplement: Barreto et al. supplementary material 2 — Barreto et al. supplementary material [file S0899823X25000893sup002.docx]

**Title:**

Getting Real Clean: A Virtual Reality Training Pilot Study for Cleaning and Low-Level Disinfection of Portable Medical Equipment.

**Authors:**

Esteban A. Barreto, PhD, MA^#,2,4^; Michelle S. Jerry, BS^#,1,5^; Vianelly García, MPH ^1,5^; Chloe V. Green, MURP^1,5^; Andrea S. Greenfield, RN ^1,5^; Eileen Searle, PhD, RN^3^; Erica S. Shenoy, MD, PhD^1,2,5,6^

1 Division of Infectious Diseases, Massachusetts General Hospital, Boston, MA

2 Harvard Medical School, Boston, MA

3 Center for Disaster Medicine, Massachusetts General Hospital, Boston, MA

4 Department of Medicine, Massachusetts General Hospital, Boston, MA

5 Infection Control, Massachusetts General Hospital, Boston, MA

6 Infection Control, Mass General Brigham, Somerville, MA

# Contributed equally.

**Supplementary Material**

**Table of Contents**

[Supplementary Table 1. 2](#_Supplementary_Table_1.)

[Supplementary Material: Virtual Reality Semi-Structured Interview Guide 2](#_Supplementary_Material:_Virtual)

[Supplementary Material: Virtual Reality Survey](#_Supplementary_Material:_Virtual_1) 6

## Supplementary Table 1.

|  | P1 N (%) | P2 N (%) |
| --- | --- | --- |
| Total | 31 (100) | 44 (100) |
| Age in years |  |  |
| 18-39 | 16 (51.6) | 20 (45.5) |
| 40 or older | 14 (45.2) | 24 (54.5) |
| Role Group |  |  |
| Infection Control/ Hospital Epidemiology | 12 (38.7) | 2 (4.6) |
| Nursing (including APRNs) | 15 (48.4) | 28 (63.6) |
| Other (e.g. Medical Assistant) | 4 (12.9) | 14 (31.8) |
| Prior VR Experience |  |  |
| No Experience | 17 (54.8) | 33 (75.0) |
| Used VR once or twice | 7 (22.6) | 8 (18.2) |
| Used VR three or more times | 7 (22.6) | 3 (6.8) |

## Supplementary Material: Virtual Reality Semi-Structured Interview Guide

Thank you for agreeing to participate in this interview and in this pilot project. The goal of our project is to evaluate the feasibility and usability of a virtual reality (VR) Infection Prevention and Control (IPC) training and assessment among diverse healthcare providers.

For this interview, we ask that you think about your experience as a virtual reality (VR) user. This interview will take approximately 30 minutes. Your responses are confidential. With your permission, we would like to record the audio of this interview to make sure that we capture everything that you share with us.

We also wanted to name that we ask the same questions of everyone, regardless of their role in the hospital, and we don’t know what job someone has until the end of the interview.

The virtual reality training that you tried out is our pilot version that we are planning to revise based on the feedback we receive. So, we want to know all the parts that worked, but also all the parts that didn’t work, or felt boring, or incorrect, or confusing, and any ideas you have to help improve it. Your honest feedback will help us revise the training and improve our VR training going forward.

Do you have any questions before we begin?

- Yes
- No

**SECTION A: PRIOR EXPERIENCE WITH VIRTUAL REALITY**

I’d like to start by asking you about your prior experience with virtual reality (VR). This could include VR video games or other training that you have been a part of.

1. Have you experienced virtual reality (VR) before?

- Yes
- No

If yes, what could you tell us about your prior experience with virtual reality? How many times have you used VR? Do you/ or anyone in your household own a VR headset?

**[PROBES]** How intuitive did you find using the controllers when navigating in the VR world? How comfortable do you feel in VR based on your prior experience? Please describe your prior experience.

______________________________________________________________________________

**SECTION B: VIRTUAL REALITY (VR) HEADSET USER EXPERIENCE**

*Now, I’d like to ask you about your experience using the VR Headset today.*

1. Which Training module did you do?

1. In general, what was it like for you to use VR?

1. How did you move around in VR (check all that apply)

- Teleporting
- Joysticks
- Walking around

1. Describe the space that you did the training in and the pros and cons of that space?

1. Were you seated or standing during the VR experience?

1. How did this experience make you feel physically? Did you experience any of the following symptoms: nausea, dizziness, headaches (from the tightness of the headset), headaches (from being in VR), eye strain or pain, other?

**[PROBES]** Did you experience any physical sensations, such as nausea or dizziness? How did your body feel?

1. What emotions did you experience?

**[PROBES**] Did you have a positive or negative emotional experience? Did you feel hypervigilant or stressed or frustrated during your time in VR (Virtual Reality)? Did you feel excited about learning this new technology?

1. Do you feel like the headset fitted properly? How did the headset fit? Did you need any assistance adjusting it during or before the training? What adjustments did you make?

1. Do you wear glasses in general and if so did you wear glasses for the VR training?

______________________________________________________________________________

**SECTION C: TROUBLESHOOTING**

Now I want to focus on your experience using the technology and any issues you might have faced.

1. What was it like for you to navigate using the controllers? Was there any action that was difficult to perform or do using the controllers.

**[PROBES]** Can you describe any challenges or issues you encountered while using the controllers?

1. Did you encounter any technical problems while using the VR Headset? If so? Did someone help you with addressing the technical problems?

**[PROBES]** What help did you need and what help did you get? Did anyone help you troubleshoot from outside the headset? What type of help did you receive? How helpful was the help that you received? Did you have any trouble connecting to the Wi-Fi or navigating to the Education XR app?

______________________________________________________________________________

**SECTION D: TRAINING SESSION**

I now want you to think about the training.

1. What do you think about this VR module as a method for teaching this topic?
2. Tell us about your previous infection prevention training? Did you like it?
3. Walk us through your favorite part of the training experience? When during the training did you feel most engaged or interested?

1. Walk us through your least favorite part of the training?
2. Did you feel confused or stuck during any part of the training?
3. At what points in the module did the person in the room with you offer tips or assistance with how to complete a step in the training? What tips did they offer?
4. What is one thing you learned in the training?
5. How did you hear about this project? What factors motivated you to sign up?
6. When you first heard about this program, what did you think?
7. Was it hard to find time to fit this into your schedule?
8. What do you think about using VR as a standard training to teach infection prevention at your (insert hospital/health center/name of institute they are from), what would make that make that possible or what would get in the way or make that hard?
9. Is there is anything else on your mind?
10. Is there something you thought we’d ask that we didn’t that you want to talk about?

 _____________________________________________________________________________

**SECTION F: ABOUT YOU** (This section was sent as a follow-up email if out of time)

Now I would like to ask some questions about you, feel free to skip any questions that you do not wish to answer.

1. What is your current role at your institution? For example, are you a Registered Nurse (RN) or Infection Preventionist (IP)?
2. How many years of experience do you have in your current role as a ____ (fill in with what they said for q18) *(If one year or less, please enter 1)*

1. In your current role, are you responsible for (check all that apply)

- Infection control
- Training other staff
- Quality and safety initiatives

1. How old are you?

1. Do you speak a language or languages other than English as your preferred or primary language when you are not at work?

- Yes
- No

**[IF YES]** What language(s) other than English, do you speak when you are not at work?

## Supplementary Material: Virtual Reality Survey

Thinking about your prior experience with VR, please answer the following questions.

1. Have you used VR before?

- Yes
- No

1. How many times have you used a VR headset?

- 1-2 times
- 3-4 times
- 5 or more times

1. Do you or someone in your household own (or have owned) a VR headset?

- Yes
- No

Thinking about your experience with the VR IPC training module, please answer the following questions.

1. Did you experience any of the following physical symptoms during or after the VR IPC Training Module? select all that apply

- dizziness
- eye strain
- headache
- nausea
- I did not experience any physical symptoms. 
  other (please specify below)

Please specify any other physical symptoms __________________

1. Did you experience any of the following emotions during the VR IPC Training Module? Check all that apply.

- excitement
- enjoyment
- frustration
- stress
- fear
- anxiety
- boredom
- curiosity
- nervousness
- other (please specify below)

Please specify any other emotions __________________ 

1. How did you move around in the VR IPC Training Module? check all that apply.

- Teleporting
- Joystick
- Walking around

1. Did you experience any glitches or technical problems during the VR IPC Training Module with any of the following? Check all that apply.

- VR Headset
- Wi-Fi
- Casting
- log in
- Controller
- battery
- other (please specify below)

Please specify any other glitches or technical issues _________________

1. Did you wear eyeglasses when participating in the VR IPC Training Module?

- Yes
- No

1. Did you have any difficulties adjusting the VR headset?

- Yes
- No

9a.  Please describe your experience trying to adjust the VR headset.

1. Did you have any difficulties using the controllers?

- Yes
- No

10a. Please describe your experience trying to use the controllers.

1. Thinking about your experience in the VR IPC Training Module, please tell us whether you agree or disagree with the following items.

|  | Strongly Disagree | Somewhat Disagree | Neutral | Somewhat Agree | Strongly Agree |
| --- | --- | --- | --- | --- | --- |
| 1. The materials and instructions I received in advance prepared me for the VR IPC Training Module. | □ | □ | □ | □ | □ |
| 1. It was easy to navigate the VR IPC Training Module using the controllers. | □ | □ | □ | □ | □ |
| 1. The virtual environment responded to my actions, movements, and the controllers the way I expected. | □ | □ | □ | □ | □ |
| 1. The virtual environment was fuzzy or blurry at times. | □ | □ | □ | □ | □ |
| 1. I was able to grab or pick up objects in the VR IPC Training Module without difficulty | □ | □ | □ | □ | □ |
| 1. It did not bother me not being aware of my surroundings | □ | □ | □ | □ | □ |
| 1. The VR IPC Training Module was more engaging than other infection control training I have taken in the past | □ | □ | □ | □ | □ |
| 1. The virtual environment was confusing* | □ | □ | □ | □ | □ |
| 1. The graphics and text were clear and easy to read | □ | □ | □ | □ | □ |
| 1. At each step, I knew what to do | □ | □ | □ | □ | □ |
| 1. My knowledge was reinforced or expanded after experiencing the VR IPC Training Module | □ | □ | □ | □ | □ |

Thinking about your experience with the VR IPC Training module, please answer the following questions.

1. Was there any part of the VR IPC Training Module that was confusing or difficult to navigate?  If yes, please explain.
2. Do you feel that you learned anything new in the VR IPC Training Module? If yes, please explain.
3. If you could change one thing about the VR IPC Training Module, what would it be?
4. Is there anything else you would like to tell us?

The following questions are about you. We would love if you could answer all the questions, however, please feel free to skip any of the questions you do not feel comfortable answering.

1. How did you hear about this program?

- Flyer
- Social Media
- Manager
- A colleague or coworker
- Email
- Other (please specify below)

1. What is your current role at your institution?

- Infection Preventionist
- Registered Nurse
- Nurse Practitioner
- Physician Assistant
- Resident
- Fellow
- Attending
- Medical Assistant
- Patient Care Technician
- Environmental Services
- Other (please specify below)

1. In your role, are you responsible for training others on infection control practices?

- Yes
- No

1. How old are you?
2. If the VR IPC Training Module was offered in a language other than English, would this be preferable to you?

- Yes
- No
